# Retraction Note: Follicle stimulating hormone modulates ovarian stem cells through alternately spliced receptor variant FSH-R3

**DOI:** 10.1186/s13048-023-01203-4

**Published:** 2023-06-21

**Authors:** Hiren Patel, Deepa Bhartiya, Seema Parte, Pranesh Gunjal, Snehal Yedurkar, Mithun Bhatt

**Affiliations:** Stem Cell Biology Department, National Institute for Research in Reproductive Health, Mumbai, 400012 India

**Retraction Note: J Ovarian Res 6, 52 (2013)**

**https://doi.org/10.1186/1757-2215-6-52**

The authors have retracted this article. After 10 years of publication, a concern was raised regarding highly similar cell pattern in different area of Fig. 2A. The corresponding author has stated that Fig. 2A is a composite image, and unintentional duplication occurred while compiling the data. The corresponding author has also stated that bright field smear images in Figs. 1–3 are composite, due to a low number of cells spread apart across the sample, which should have been indicated in the figure legends. The authors are preparing a revised version of this manuscript for peer review.

Hiren Patel, Deepa Bhartiya, Seema Parte and Mithun Bhatt agree to this retraction. The Publisher has not been able to obtain current email addresses for Pranesh Gunjal and Snehal Yedurkar.

